# TMEM160 Promotes Tumor Growth in Lung Adenocarcinoma and Cervical Adenocarcinoma Cell Lines

**DOI:** 10.3390/ijms26031097

**Published:** 2025-01-27

**Authors:** Gloria Angelina Herrera-Quiterio, Heriberto Abraham Valencia-González, Karen Griselda de la Cruz-López, Diana Lashidua Fernández-Coto, Jeovanis Gil, György Marko-Varga, Josué Morales-Gálvez, Nilda C. Sánchez, Rubén Rodríguez-Bautista, Alejandro Avilés-Salas, Oscar Arrieta, Alejandro García-Carrancá, Sergio Encarnación-Guevara

**Affiliations:** 1 Programa de Doctorado en Ciencias Bioquímicas, Centro de Ciencias Genómicas, Universidad Nacional Autónoma de México, Cuernavaca 62210, Morelos, Mexico; gloriaangelinahq@gmail.com; 2 Laboratorio de Proteómica, Centro de Ciencias Genómicas, Universidad Nacional Autónoma de México, Cuernavaca 62210, Morelos, Mexico; cocodi_trent@hotmail.com (D.L.F.-C.); josueemg01@gmail.com (J.M.-G.); nildita1985@gmail.com (N.C.S.); 3 Laboratorio Virus y Cáncer, Instituto Nacional de Cancerología, Mexico City 14080, CDMX, Mexico; valenciabraham@gmail.com; 4 Posgrado en Ciencias Biomédicas, Instituto de Investigaciones Biomédicas, Universidad Nacional Autónoma de México, Mexico City 04510, CDMX, Mexico; karencl_1991@hotmail.com; 5 Clinical Chemistry Section, Department of Translational Medicine, Lund University, 22242 Lund, Sweden; jeovanis.gilvaldes@med.lu.se; 6 Clinical Protein Science & Imaging, Department of Biomedical Engineering, Lund University, 22242 Lund, Sweden; gyorgy.marko-varga@bme.lth.se; 7 Breast Cancer Unit, National Cancer Institute, Mexico City 14080, CDMX, Mexico; rubenrb@comunidad.unam.mx; 8 Pathology Department, National Cancer Institute, Mexico City 14080, CDMX, Mexico; alejandroaviles2001@yahoo.com (A.A.-S.); ogarrieta@gmail.com (O.A.); 9 Thoracic Oncology Unit, National Cancer Institute, Mexico City 14080, CDMX, Mexico; 10 Unidad de Investigación Biomédica en Cáncer, Instituto de Investigaciones Biomédicas, Universidad Nacional Autónoma de México & Instituto Nacional de Cancerología, Mexico City 04510, CDMX, Mexico; carranca@biomedicas.unam.mx

**Keywords:** C-HPP, TMEM160, non-small cell lung cancer, cervical cancer, tumor growth, nuclear localization

## Abstract

The Chromosome-Centric Human Proteome Project (C-HPP) is an international initiative. It aims to create a protein list expressed in human cells by each chromosomal and mitochondrial DNA to enhance our understanding of disease mechanisms, akin to the gene list generated by the Human Genome Project. Transmembrane protein 160 (TMEM160) is a member of the transmembrane proteins (TMEM) family. TMEM proteins have been implicated in cancer-related processes, including cell proliferation, migration, epithelial-mesenchymal transition, metastasis, and resistance to chemotherapy and radiotherapy. This study aimed to investigate the role of TMEM160 in non-small cell lung cancer and cervical cancer using cell lines, clinical samples, and xenograft studies. Our findings demonstrated that TMEM160 knockdown decreased the proliferation of lung and cervical cancer cell lines. We observed that TMEM160 is localized in the nucleus and cytoplasm and dynamic localization during mitosis of cancer cells and discovered a novel interaction between TMEM160 and nuclear proteins such as NUP50. Furthermore, the TMEM160 interactome was enriched in processes associated with apical junctions, xenobiotic metabolism, glycolysis, epithelial-mesenchymal transition, reactive oxygen species, UV response DNA, the P53 pathway, and the mitotic spindle. This study provides an initial understanding of the function of TMEM160 in lung and cervical cancer progression and clarifies the need to continue investigating the participation of TMEM160 in these cancers.

## 1. Introduction

Lung and cervical cancers are among the most common cancers worldwide [1]. Lung cancer is the most prevalent cancer in men and the second most prevalent in women [1]. There are two main types of lung cancer: small-cell lung cancer (SCLC) and non-small-cell lung cancer (NSCLC). NSCLC accounts for 85% of cases [2]. NSCLC is further classified into three histological subtypes: large cell carcinoma, squamous cell carcinoma, and adenocarcinoma (LUAD), with LUAD being the most prevalent subtype, accounting for 40% of the cases [3]. The main issues in LUAD are tumor heterogeneity due to the diversity of genetic variants within the same type of tumor and the low five-year survival rate for LUAD, ranging between 2% and 20% [4]. Therefore, it is essential to investigate the pathogenesis of LUAD and to identify potential therapeutic targets.

Cervical cancer (CC) ranks fourth in terms of incidence and mortality among gynecological neoplasms worldwide [1]. The two primary histological subtypes of cervical cancer are adenocarcinoma and squamous cell carcinoma, which together account for approximately 92% of the cases. The remaining 8% of cases consist of less common subtypes: adenosquamous carcinoma, neuroendocrine carcinoma, and undifferentiated carcinoma [5,6]. Although there are prevention schemes through HPV vaccination and early detection, the quality of life of patients remains limited [7]. Therefore, it is vital to investigate the molecular mechanisms driving the progression of cervical cancer.

The Chromosome-Centric Human Proteome Project (C-HPP) is an international initiative by the Human Proteome Organization (HUPO). It aims to identify, quantify, and localize proteins expressed in human cells from each chromosome. The goal is to create a comprehensive list of proteins to enhance our understanding of cellular functions and diseases [8,9].

In many types of cancer, transmembrane protein family (TMEM) proteins are oncogenes that promote proliferation, migration, invasion, epithelial-mesenchymal transition (EMT), metastasis, and resistance to chemotherapy and radiotherapy [10,11,12,13,14]. In contrast, other proteins of the TMEM family are associated with tumor suppressor functions [15,16,17,18,19]. Some TMEM proteins have been reported to exert both functions in different types of cancer [20,21,22,23,24,25].

While TMEM proteins were initially described as membrane-associated, studies have shown that some members of this family are also located in other cellular compartments. Apparently, the localization of TMEM proteins is not limited to biological membranes, as some are found in the cytosol and secreted outside the cell, such as TMEM88 and TMEM98, respectively, in addition to being located in the plasma membrane [21,26].

The protein TMEM160 regulates the generation of reactive oxygen species and the unfolded protein response in mitochondria [27]. Recently, TMEM160 was linked to immune evasion in tumors and resistance to radiotherapy in colorectal cancer [28]. However, the role of TMEM160 in lung and cervical cancers has not been elucidated. In this study, we investigated the role of TMEM160 in lung adenocarcinoma (LUAD) and cervical cancer. We identified a novel interaction between TMEM160 and the nuclear protein NUP50, a nucleoporin that binds directly to importin, in A549 cells. This interaction has not been previously described. In addition, we observed nuclear and cytoplasmic localization of TMEM160 in both tumor and non-tumor cells of the lung and cervix. Also, we discovered that the knockdown of TMEM160 decreased cell proliferation and migration in vitro and decreased tumor growth in vivo. Our study highlighted the biological functions of TMEM160 in LUAD and cervical cancer and suggested its potential value as a therapeutic target for LUAD and cervical cancer treatment.

## 2. Results

### 2.1. TMEM160 Is Localized in the Nucleus and Cytoplasm of the Tumor and Non-Tumor Cells of the Lung and Cervix

Initial reports showed that overexpression of TMEM160 in HEK293 and HeLa cells resulted in its localization to the inner membrane of the mitochondria [27]. However, when overexpressed in HCT116 cells, it was found in the cytoplasm and cell membrane [28]. We investigated the subcellular localization of endogenous TMEM160 in various lung and cervical cancer cell lines (A549, H1975, HeLa, and SiHa) and corresponding non-tumor cell lines (BEAS and HaCaT). First, to assess the antibody specificity, Western blot analysis revealed a single band corresponding to the expected molecular weight of TMEM160 in both HaCaT and HeLa cells (Figure S1), thereby supporting the accuracy of our localization data.

**Figure 1:**
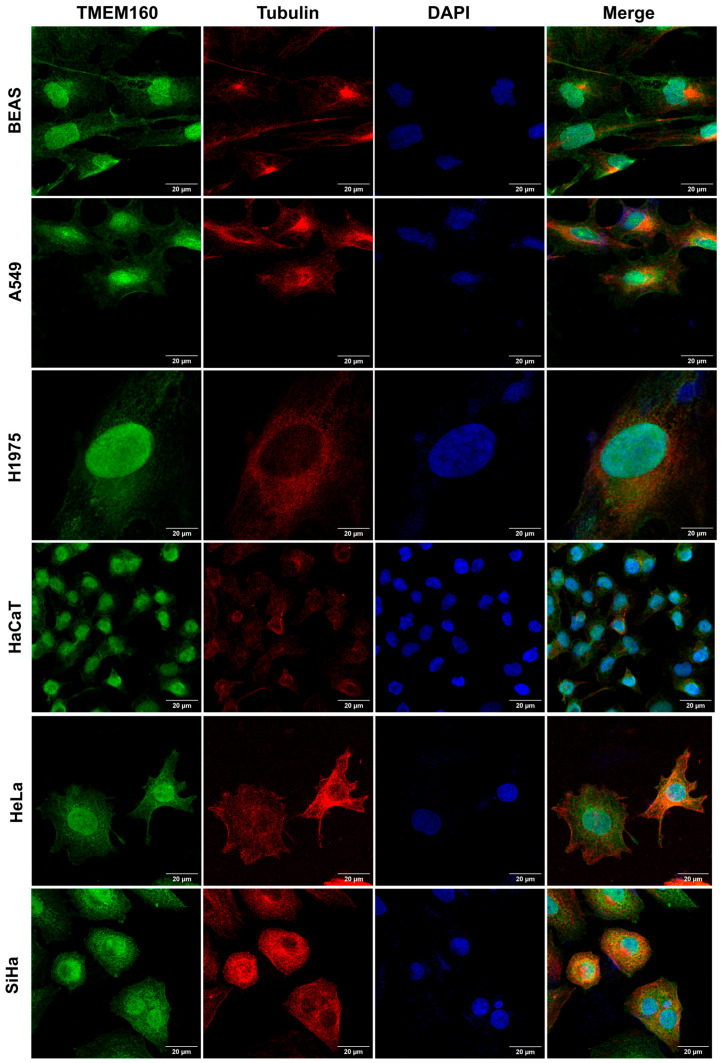
TMEM160 localization in the nucleus and cytoplasm of lung and cervix tumor and non-tumor cell lines. BEAS, A549, H1975, HaCaT, HeLa, and SiHa were stained, and images clearly indicate that TMEM160 (green) is localized both in the nucleus and cytoplasm of the cells, as evidenced by its co-localization with tubulin (red) in the cytoplasm and DAPI (blue) in the nucleus. Scale bar: 20 µm. Images were acquired using a 60× objective with 2× zoom.

**Figure 2:**
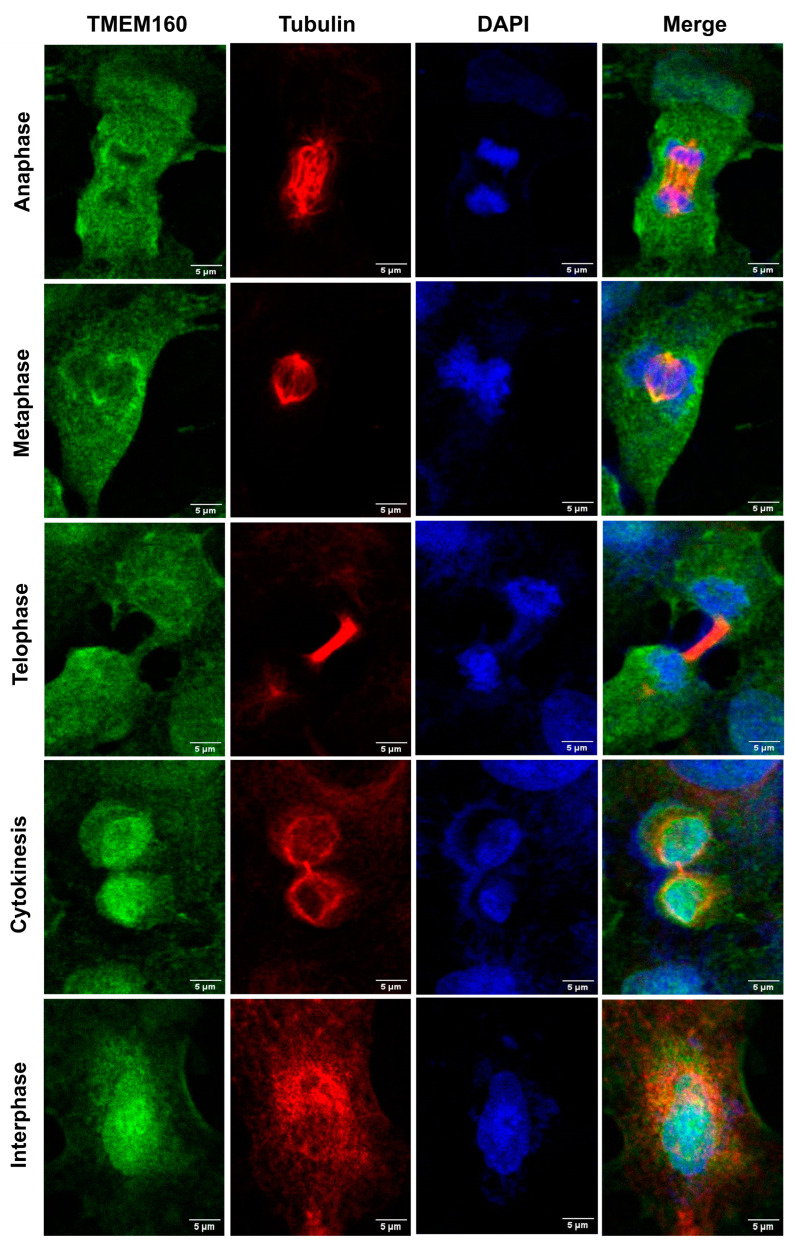
TMEM160 shows dynamic localization during cell division. TMEM160 signal in A549 cells at different mitotic stages. TMEM160 is primarily located in the cytoplasm during metaphase, anaphase, and telophase, with the nuclei lacking signal in these phases. During cytokinesis, the signal reappears in the nucleus, while in interphase cells the signal of TMEM160 is located in both the nucleus and the cytoplasm. Scale bar: 20 µm. Images were acquired using a 60× objective with 2× zoom.

Immunostaining of BEAS, A549, H1975, HaCaT, HeLa, and SiHa cells showed that TMEM160 was present predominantly in the nucleus, with a lesser extent of the signal observed in the cytoplasm in all tested cell lines (Figure 1). The cytoplasmic localization was further supported by colocalization with tubulin, quantified using the Manders coefficients (Table S1). Notably, TMEM160 showed dynamic localization patterns, with predominant cytoplasmic distribution during cell division, particularly in mitotic cells. During anaphase, metaphase, and telophase, TMEM160 signals were predominantly shifted to the cytoplasm, and chromosomes were largely devoid of TMEM160 signals. However, in cytokinesis, a shift occurred as TMEM160 signals accumulated in chromosomes while still present in the cytoplasm. In interphase cells, all TMEM160 signals were found primarily in the nucleus and, to a lesser extent, in the cytoplasm, overlapping with DAPI and tubulin signals. These results underscore the dynamic nature of TMEM160 subcellular localization during the cell cycle (Figure 2). Primary negative controls (Figure S2), cross-emission controls (Figure S3), and autofluorescence controls (Figure S4) support our findings. They also reveal a novel aspect of TMEM160 biology, showing that endogenous TMEM160 is dynamically localized in both the nucleus and cytoplasm, and this could indicate that TMEM160 carries out a function in mitotic events of tumor and non-tumor cervical and lung cell lines.

### 2.2. Confirmation of Nuclear and Cytoplasmatic Localization of TMEM160

**Figure 3:**
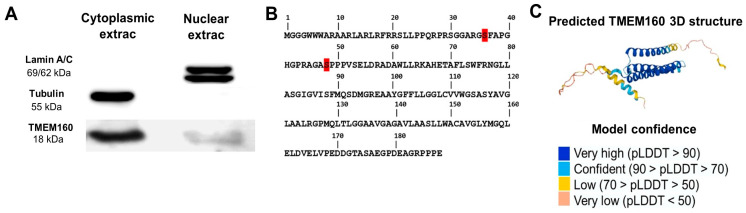
Confirmation of the nuclear and cytoplasmic localization of TMEM160. (**A**) Western blot analysis of cell fractionation from A549. The nucleus-resident lamin A protein, cytoplasm-resident tubulin, and TMEM160 were detected in both fractions. The entire volume of the nuclear fraction, along with an equivalent volume of the cytoplasmic fraction, were loaded onto the electrophoresis gel. This reflects differences in total protein content in the Western blot, but not the relative abundance of TMEM160 between cell fractions. (**B**) TMEM160 has two phosphorylation residues at Ser36 and Ser48 that can be phosphorylated. (**C**) Predicted structure of TMEM160, according to Uniprot, is provided with high confidence; two disordered regions at both the N and C terminus are identified.

To confirm the localization of TMEM160 in the nucleus and cytoplasm, we conducted cell fractionation using the NE-PER Nuclear and Cytoplasmic Extraction Kit in A549 cells. Western blot analysis was used to detect lamin A/C, proteins found exclusively in the nucleus, and tubulin, a cytoplasmic protein, to evaluate fraction purity. We observed TMEM160 in both the nuclear and cytoplasmic fractions. It is important to note that this experiment confirms the presence of TMEM160 in both compartments but does not provide a quantitative comparison of its abundance (Figure 3A). These findings confirm the nuclear and cytoplasmic localization of endogenous TMEM160.

Furthermore, we investigated in databases to see if TMEM160 contains a nuclear localization signal (NLS) or other domains participating in its nuclear targeting: SignalP-6.0 (https://services.healthtech.dtu.dk/services/SignalP-6.0/, accessed on 15 June 2024), InterPro (www.ebi.ac.uk/interpro/, accessed on 15 June 2024), DeepLoc (https://services.healthtech.dtu.dk/services/DeepLoc-2.1/, accessed on 15 June 2024). No NLS was identified in TMEM160. However, in the PhosphoSite database (https://www.phosphosite.org/homeAction, accessed on 15 June 2024), we identified two phosphorylation sites in the TMEM160 sequence at serine residues 36 and 48 (Figure 3B). Additionally, the predicted structure for TMEM160 available in UniProtKB, which demonstrated satisfactory quality throughout the template on matching and transmembrane domain analysis, reveals two disordered regions (Figure 3C). Disordered regions have been reported to enable proteins to interact with DNA [29]. Based on these results, it is possible that the phosphorylation sites of TMEM160 are involved in its translocation to the nucleus, and its disordered domains are related to its function in the nucleus. However, further studies are needed to determine whether the phosphorylation of serine residues 36 and 48 in TMEM160 affects its subcellular localization in the nucleus.

### 2.3. TMEM160 Is Upregulated in LUAD and Cervical Cancer

**Figure 4:**
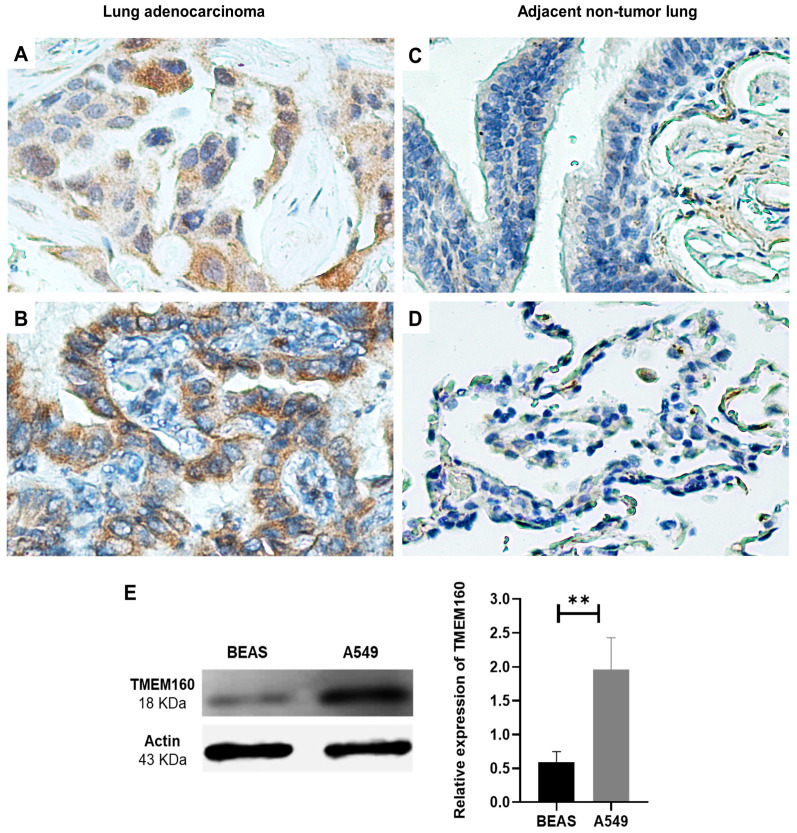
TMEM160 is upregulated in LUAD and cervical cancer. (**A**–**D**) TMEM160 protein levels were evaluated in clinical samples, with six lung cancer samples confirmed as adenocarcinomas. (**A**,**B**) Representative images of two adenocarcinoma tissue samples showing a strong TMEM160 signal compared to the adjacent non-tumor tissue without signal (**C**,**D**). (**E**) TMEM160 expression was significantly higher in A549 cell line compared to the non-tumor BEAS cell line (*p* = 0.008). Actin was used as a loading control to ensure uniform protein loading across all samples. ** *p* < 0.01.

First, we analyzed immunohistochemistry data of The Human Protein Atlas. We found that the intense staining indicates a high expression level of TMEM160 in lung adenocarcinoma tissues (Figure S5A), as well as in adenocarcinoma and squamous cell carcinoma cervical tissues (Figure S5C). In contrast, the signal was weak in non-tumor lung and cervical tissues (Figure S5B–D). We then conducted an immunohistochemical analysis with six clinical samples of LUAD. Our results showed that TMEM160 protein exhibited a median expression HR score of 120 (range 50–247.5), with predominant localization in the cytoplasm of the cells, while in the adjacent non-tumor tissue, the signal for TMEM160 was negative (Figure 4A–D). The patient clinical characteristics are listed in Table S2.

We also consulted the Expression Atlas (https://www.ebi.ac.uk/gxa/about.html, accessed on 19 January 2021) to identify the proteomic data for TMEM160. The data revealed that both A549 and HeLa cell lines had high levels of TMEM160 in two independent proteomic studies (Figure S2E,F) [30,31]. Additionally, we assessed the expression level of TMEM160 in the A549 and BEAS cell lines. In Western blots, TMEM160 expression was significantly higher in the A549 cell line compared to the BEAS cell line (*p* = 0.008) (Figure 4E). These results suggest that overexpression of TMEM160 is upregulated in LUAD and cervical cancer and may play a crucial role in these cancers.

### 2.4. The Interactome of TMEM160 Is Related to Biological Processes and Protumorigenic Pathways Associated with Nuclear Proteins, DNA Replication, and the Cell Cycle in LUAD

**Figure 5:**
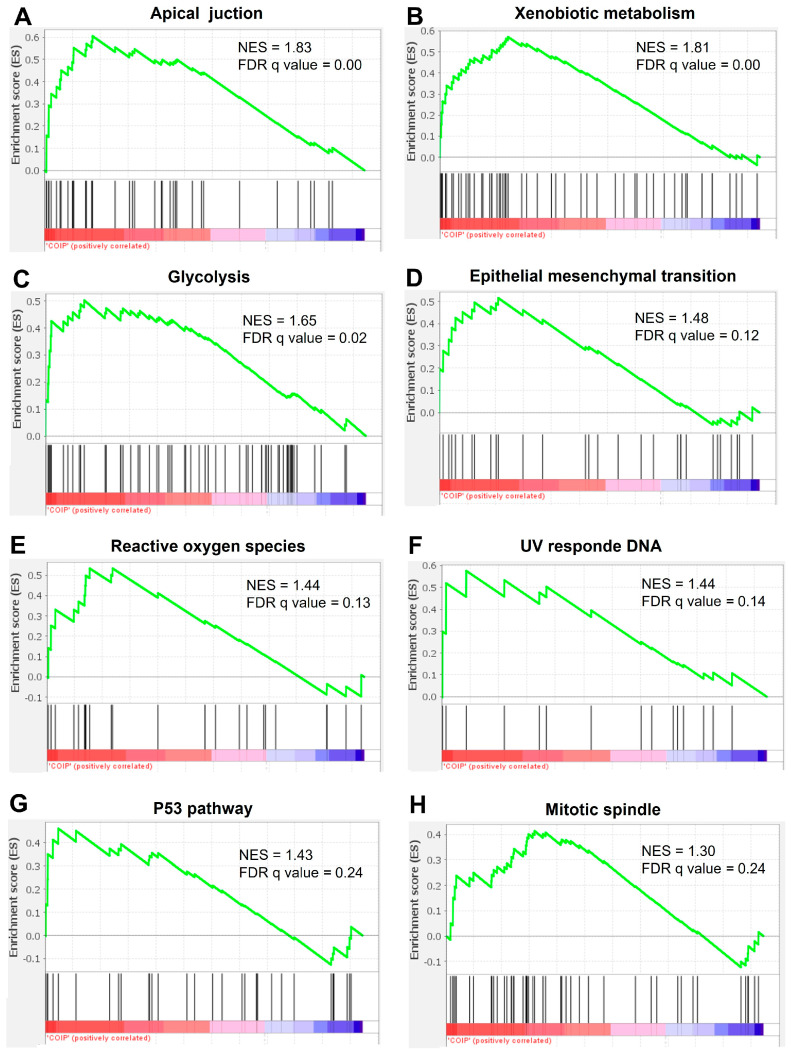
TMEM160 is related to protumorigenic pathways in LUAD. The GSEA analysis of proteins identified in Co-Ip of A549 cells reveals an association of TMEM160 with the following pathways: (**A**) apical junction, (**B**) xenobiotic metabolism, (**C**) glycolysis, (**D**) EMT, (**E**) reactive oxygen species, (**F**) UV response DNA, (**G**) P53 pathway, and (**H**) mitotic spindle. The color bar below each plot indicates the correlation of genes with Co-IP condition, where red represents a positive correlation and blue a negative correlation. NES: Normalized enrichment score; FDR: False discovery rate q-value.

To elucidate the role of TMEM160 in lung adenocarcinoma (LUAD), we performed co-immunoprecipitation (Co-IP) followed by mass spectrometry (LC-MS/MS) to identify proteins interacting with TMEM160 in the A549 cell line. Western blotting confirmed the successful enrichment of TMEM160 in immunoprecipitates (Figure S6). After filtering and normalizing the Co-IP and LC-MS/MS data, we conducted gene set enrichment analysis (GSEA) on the TMEM160 interactome proteins. Based on its normalized enrichment score, this analysis revealed a complex interactome associated with several cancer-related pathways and biological processes. These are apical junctions, xenobiotic metabolism, glycolysis, EMT, reactive oxygen species, UV response DNA, the P53 pathway, and the mitotic spindle (Figure 5A–H). These findings suggest that TMEM160 may be involved in the pathogenesis and progression of LUAD.

**Figure 6:**
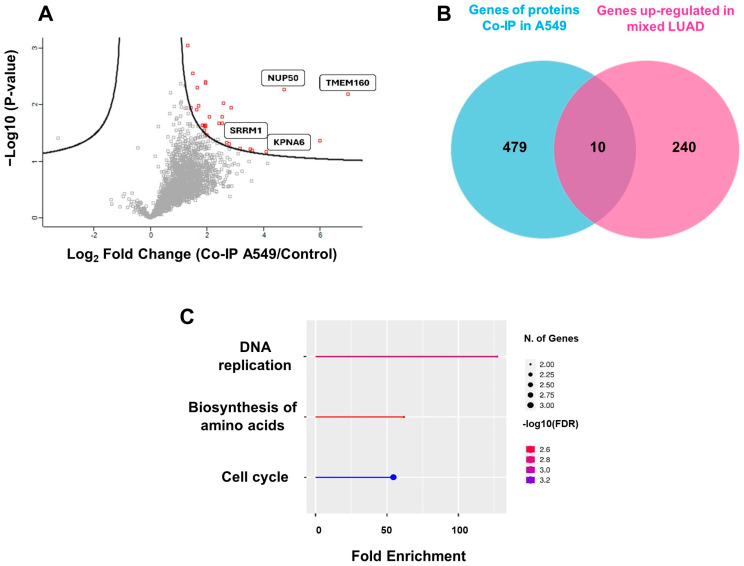
The interactome of TMEM160 is associated with nuclear proteins, DNA replication, and the cell cycle. (**A**) Volcano plot representing the significant differences in protein concentration values between the Co-Ip A549 and the negative control of Co-Ip. The X-axis shows the log2 transformation of the fold change between the Co-Ip A549 and the negative control. The Y-axis represents the -log10 transformation of the *p*-values for each protein. Red dots indicate proteins with significant changes. (**B**) In the Co-IP samples, 480 unique genes were identified, and it was found that the 250 most commonly upregulated genes in LUAD were also found in the UALCAN database. A comparison of these two groups revealed 10 shared proteins. (**C**) Significantly enriched biological process GO terms (FDR < 0.05) of the 10 proteins common between Co-Ip and LUAD, which are involved in DNA replication, amino acid synthesis, and cell cycle.

In the TMEM160 interactome, 31 proteins were significantly enriched, showing fold changes greater than 2.0 and up to 6.5 compared to the control group (Figure 6A and Table S3). To further explore the functional annotations of these 31 proteins, a Gene Ontology biological processes enrichment analysis was performed based on their pathways. This analysis revealed that the significantly enriched biological processes were nucleocytoplasmic transport, involving the nucleoporin NUP50, importin KPNA6, and SRRM1 (Figure 6A). These findings suggest that the function of TMEM160 is related to a process regulated from the nucleus, although the underlying function remains unclear.

Figure 6 shows that the interactome of TMEM160 is related to nuclear proteins, DNA replication, and the cell cycle. Specifically, 489 proteins/genes exclusive to the TMEM160 interactome were identified compared to those in the control group (Table S4). The UALCAN database (https://ualcan.path.uab.edu/index.html, accessed on 4 April 2024) was used to identify 250 genes that were commonly upregulated in mixed subtype LUAD (Table S5). Furthermore, a Venn diagram showed that 10 genes were shared between 489 proteins and 250 genes (Figure 6B). GO enrichment analysis was conducted to investigate the functional annotations of these 10 genes, focusing on pathways. The significantly enriched pathways identified were DNA replication, amino acid biosynthesis, and cell cycle (Figure 6C). These data indicate that TMEM160 interacts with proteins upregulated in LUAD, suggesting that TMEM160 is involved in the biogenesis and progression of this pathology.

### 2.5. Knockout of TMEM160 Inhibits the Proliferation and Migration of LUAD Cells In Vitro

**Figure 7:**
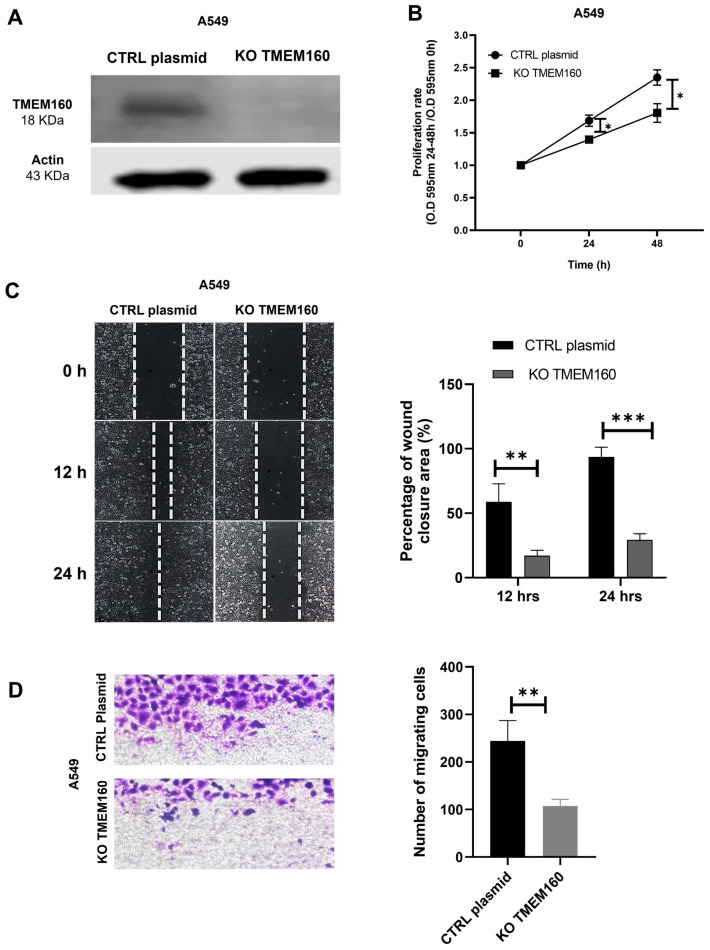
Knockout of TMEM160 inhibits the proliferation and migration of lung cancer cells in vitro. (**A**) Western blotting demonstrated that CRISPR/CAS transfection efficiently eliminated TMEM160 in A549 cells. Actin was used as a loading control to ensure uniform protein loading across all samples. (**B**) TMEM160 significantly led to a significant decrease in proliferation at 12 h (*p* < 0.0047) and 24 h (*p* < 0.0068). (**C**) In a wound closure assay, it was observed that TMEM160 knockdown significantly decreased wound closure at 12 h (*p* < 0.007) and 24 h (*p* < 0.0002). (**D**) Migration assay results showed that knockdown of TMEM160 reduced the migration capacity of A549 cells (*p* < 0.0065). All values are presented as mean ± SD. All experiments were conducted at least three times. * *p* < 0.05, ** *p* < 0.01, *** *p* < 0.001.

To substantiate the potential role of TMEM160 in the pathogenesis and progression of lung cancer, we used the CRISPR/Cas9 system to silence the expression of TMEM160 in A549 cells (KO TMEM160) (Figure 7A). Control cells were transfected with a non-targeting plasmid (CTRL plasmid). We assessed cell proliferation in vitro using the MTT assay and found that KO TMEM160 significantly reduced cell proliferation at 24 and 48 h compared to control cells A549 (Figure 7B). Furthermore, our wound closure assay demonstrated that silencing KO TMEM160 notably slowed the rate of wound closure in A549 cells at 12 and 24 h (Figure 7C). Similarly, the transwell migration assay showed that KO TMEM160 significantly decreased the migratory ability of A549 KO TMEM160 cells (Figure 7D). These findings strongly indicate that the knockout of TMEM160 decreases the proliferation and migration of lung cancer cells.

### 2.6. Silencing TMEM160 Inhibits Tumor Growth of LUAD and Cervical Cancer Cells In Vivo

To confirm the effect of TMEM160 on tumor growth in vivo, we conducted animal experiments in immunodeficient NOD-SCID mice. Initially, we created a knockout clone of TMEM160 in A549 cells, and the expression of TMEM160 protein recovered over time. Therefore, before performing the xenograft, the cells were sorted using a cytometer and evaluated using Western blotting, resulting in a knockdown clone (KD TMEM160).

**Figure 8:**
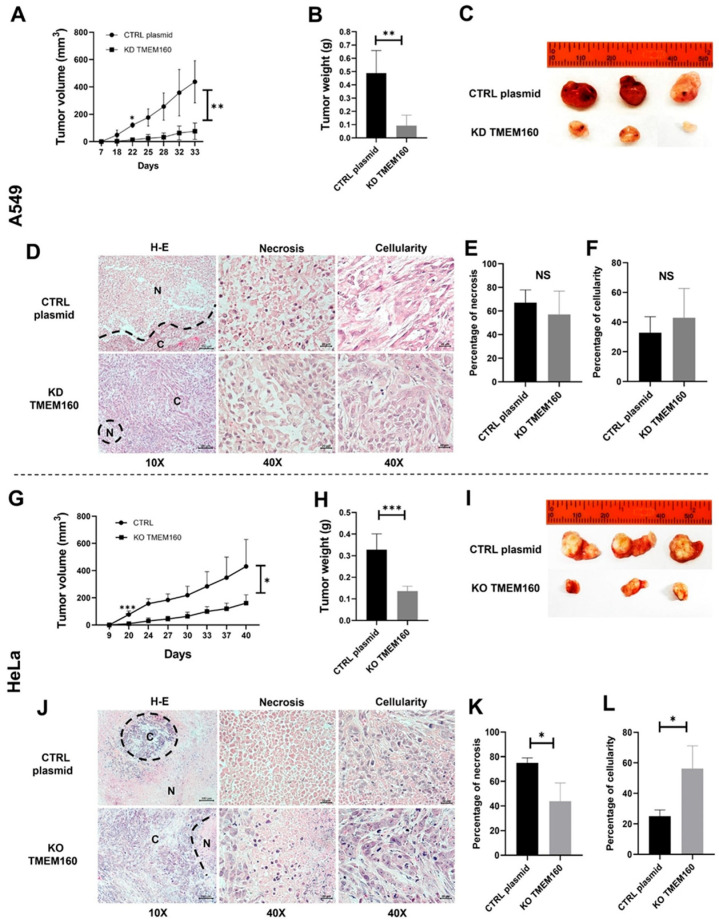
Silencing TMEM160 reduces tumor growth in LUAD and cervical cancer cells in vivo. (**A**–**F**) Xenotransplantation in A549 cells. (**A**) Growth curve and (**B**) mean weight of xenograft tumors in NOD SCID mice were measured for 33 days. (**C**) Representative images of subcutaneous xenograft tumors from NOD SCID mice in each group. (**D**) Hematoxylin-eosin (H-E) staining in the CTRL group and the KD TMEM160 group. (**E**) Tendency for greater necrosis in the CTRL group. (**F**) Greater cellularity in the KD TMEM160 group compared to the CTRL group. (**G**–**L**) Xenotransplantation in HeLa cells. (**G**) Growth curve and (**H**) mean weight of xenograft tumors in NOD SCID mice were measured for 40 days. (**I**) Representative images of subcutaneous xenograft tumors from NOD SCID mice in each group. (**J**) Tumor section H-E staining in the CTRL group and KD TMEM160. (**K**) Greater necrosis in the CTRL group (*p* = 0.0286). (**L**) Reduce cellularity in the CTRL group compared to the KD TMEM160 group. Showing three of five xenografts. (* *p* < 0.05, ** *p* < 0.01, *** *p* < 0.001, NS: non-significant).

We confirmed that reduced TMEM160 expression attenuated LUAD proliferation by monitoring the xenograft growth curve. Significant changes were observed from day 18 (*p* = 0.04) and continued until the end of the experiment on day 33 (*p* = 0.004) (Figure 8A). Xenografts established from A549 KD TMEM160 cells exhibited significantly lower tumor weights (*p* = 0.0058) and smaller tumor sizes than those established from A549 CTRL plasmid cells (Figure 8B,C). H&E staining revealed a tendency for greater necrosis in the CTRL group compared to the A549 KD TMEM160 cell xenografts (Figure 8D,E).

Consistent with the necrosis in the KD TMEM160 group, the percentage of cellularity showed a tendency for greater cellularity compared to the CTRL group (Figure 8D). In contrast, the CTRL plasmid group exhibited faster tumor growth, as indicated by tumor volume, which likely limited nutrient access and subsequently induced necrosis.

As the TMEM160 protein was initially identified as being overexpressed in HeLa cells, we evaluated whether TMEM160 knockout in HeLa cells would affect tumor growth. The results demonstrated that TMEM160 knockout significantly reduced the tumor volume from day 20 (*p* = 0.0006) and continued through the end of the experiment on day 40 (*p* = 0.01) (Figure 8G) and weight (*p* = 0.0005) in HeLa cell xenografts in female NOD SCID mice (Figure 8H,I). Similar to the A549 KD TMEM160 xenografts, H&E staining revealed fewer necrotic areas in Hela KD TMEM160 cell xenografts compared to the CTRL plasmid cells (*p* = 0.0286) (Figure 8J,K). Consistent with this observation, the percentage of cellularity was higher in xenografts from HeLa KO TMEM160 cells (*p* = 0.0286) (Figure 8L). These findings highlight the relationship between necrosis, cellularity, and tumor growth, confirming that TMEM160 promotes tumor growth progression in both LUAD and cervical cancer.

## 3. Discussion

The C-HPP consortium comprises 25 international research teams. Within this consortium, 24 chromosomes and mitochondrial DNA were assigned for analysis. The main goal of the C-HPP is to identify each canonical protein accurately, characterize the main proteoforms, elucidate the functions of proteins encoded by each chromosome that remain functionally unassigned, and clarify their roles in human cells [8,9]. Among these proteins is TMEM160, encoded by human chromosome 19 [32].

TMEM family proteins are located in various cellular membranes [33,34]. A key finding of our study is the first-time demonstration that endogenous TMEM160 is localized predominantly in the nucleus and, to a lesser extent, in the cytoplasm of lung and cervical tumor and non-tumor cell lines, displaying dynamic localization patterns during cell division. This suggests that TMEM160 has a role in mitotic events, probably through interaction with cytoskeletal proteins; however, further studies are needed to directly establish the role of TMEM160 in mitotic events. Another important observation is that while TMEM proteins were initially described as membrane-associated, our findings support the observation that TMEM proteins are not exclusively localized in the membranes. For instance, TMEM98, a type II transmembrane protein secreted via the exosome pathway [26]. Similarly, TMEM88, primarily located in the plasma membrane, is also present in the cytoplasm and, to a lesser extent, in the nucleus [21], which indicates that TMEM proteins have roles that go beyond membrane functions.

Protein import into the nucleus is highly regulated and is mediated by the nuclear pore complex (NPC) [35]. Our analysis of the primary amino acid sequence of TMEM160 revealed two serine residues, Ser36 and Ser48, that are susceptible to phosphorylation. This finding is significant because phosphorylation is a posttranslational modification that regulates the translocation of a wide range of proteins into the nucleus [36,37]. For example, NF-κB, a cytoplasmic protein, is phosphorylated on serine 536 of the p65 subunit, facilitating its translocation to the nucleus [38]. Alterations in phosphorylation pathways can lead to continuous activation of cellular processes and have been linked to different types of cancer [36,39]. Although no nuclear localization signal (NLS) was identified in TMEM160, the phosphorylation of Ser36 and Ser48 may influence its translocation to the nucleus through a mechanism that is not fully understood. Further studies are necessary to determine if the phosphorylation of these serine residues affects the subcellular localization of TMEM160. Additionally, proteins with molecular weights less than approximately 40 to 60 kDa can enter the nucleus through passive diffusion across the NPC without requiring an NLS [40]. With a molecular weight of 18 kDa [32], the nuclear import of TMEM160 likely occurs via passive diffusion, highlighting the need for further investigation into this mechanism.

The predicted structure of TMEM160 in the Uniprot database shows disordered regions in both the N-terminal and C-terminal regions [32]. These disordered regions also referred to as unstructured regions, represent segments of proteins that do not adopt a fixed three-dimensional conformation, unlike regions that form stable secondary structures such as alpha helices and beta sheets [41,42]. Disordered regions of proteins are involved in various biological functions, including cellular signaling, transcription regulation, and the cell cycle, and facilitate protein-peptide interactions with multiple partners [43]. Moreover, these regions act as affinity modulators in DNA-protein interactions [29]. Consequently, when TMEM160 localizes to the nucleus, it may bind to DNA through its disordered regions.

TMEM family proteins are expressed in many cell types and are involved in numerous biological functions, including immune response [26,44], protein glycosylation [45], regulation of autophagy [46], cell differentiation [47,48], and epidermal morphogenesis [49]. Moreover, TMEM proteins have been linked to both oncogenic and tumor-suppressive roles in different types of cancer [33,34,50]. However, the specific expression and function of TMEM160 have not been studied in lung and cervical cancers.

Our study demonstrates that TMEM160 is significantly upregulated in lung adenocarcinoma and cervical cancer, where it is found in both the cytoplasm and nucleus of cancer cells.

These findings suggest that TMEM160 may interact directly with DNA; specifically, our proteomic data from Co-Ip indicated that TMEM160 is associated with nuclear proteins such as nucleoporin NUP50, importin KPNA6, and SRm1. NUP50 is a component of the nuclear pore complex (NPC) that regulates the bidirectional transport of molecules between the nucleus and cytoplasm in eukaryotic cells [51]. It is also localized to the nucleoplasm, participates in gene regulation [52], and is associated with DNA damage repair [53]. In *Caenorhabditis elegans*, NUP50 plays a role in the cell cycle checkpoint function [54]. Importin KPNA6 is involved in transporting proteins into the nucleus and acts as an adapter protein for the nuclear receptor KPNB1, directly binding to substrates containing simple or bipartite NLS [35]. KPNA6 may promote breast cancer cell proliferation [55]. Meanwhile, SRRM1 is involved in alternative splicing, producing various protein isoforms from a single gene [56]. The significant interaction of nuclear proteins and TMEM160 aligns with their nuclear localization and suggests potential functions that favor cancer cells.

Our interactome analysis indicates that TMEM160 is associated with multiple cancer-related pathways, including those involved in apical junction, xenobiotic metabolism, glycolysis, EMT, mitotic spindle reactive oxygen species, UV response DNA, and the P53 pathway. EMT is a well-documented cellular process that plays a key role in tumor cell behavior, enhancing their ability to invade adjacent tissues and consequently form metastases [50,57]. The association of TMEM160 with EMT suggests it may have metastatic potential. Additionally, previous studies have shown that knocking down TMEM160 increases oxidative stress by triggering a mitochondrial unfolded protein response (UPRmt) and elevating ROS production [27]. Our findings emphasize the ROS-related pathways, indicating that TMEM160 may be linked to cellular stress responses, enabling cancer cells to survive in hostile environments. It has been documented that TMEM160 promotes radiotherapy resistance in colorectal cancer cells through immune system evasion [28]. We also found that the TMEM160 interactome is associated with xenobiotic metabolism, closely related to chemoresistance in cancer cells. This process influences the ability of tumor cells to metabolize cytotoxic agents through various mechanisms, including the activity of carrier proteins that promote drug efflux, inactivation of therapeutic compounds, participation in DNA damage repair, and the action of antioxidant enzymes, among others [58,59]. Therefore, further studies are necessary to investigate the involvement of TMEM160 in the response to chemotherapy.

In summary, we report here that the expression of TMEM160 is upregulated in lung cancer and cervical cancer cells. Given the interactions and processes described in this study, we now understand that TMEM160 is associated with the P53 pathway, mitotic spindle, DNA replication, and cell cycle processes, indicating its involvement in cell proliferation, tumor growth, and tumor dynamics. Although the exact mechanism remains unknown, our findings suggest that further investigation is needed to determine how TMEM160 influences tumor progression and promotes the growth of tumor cells in LUAD and cervical cancer.

## 4. Materials and Methods

### 4.1. Patients and Tissue Samples

This study was approved by the Research and Ethics Committee at the National Cancer Institute (INCan). Six NSCLN tissues and adjacent non-tumor lung tissues were collected from patients. These tissues were embedded in paraffin blocks and were part of the INCan pathology service. The slides used were previously consented to by the patients. Tissues fixed in 4% paraformaldehyde and embedded in paraffin blocks were cut into layers up to 3 mm thick to be placed on loaded slides for immunohistochemical staining of TMEM160 protein.

### 4.2. Immunohistochemistry (IHQ)

IHQ was performed to detect TMEM160 protein expression in lung cancer and adjacent non-tumor lung tissues. Tissues were deparaffinized at 60 °C for one hour. For antigen retrieval, the slides were immersed in 10 mM sodium citrate buffer solution (pH 6) and heated in a pressure cooker for 15 min. The slides were incubated with an endogenous peroxidase blocking solution from the Mohs mouse/rabbit brown PoliDetector DAB HRP kit (BioSB) for 15 min, twice. Subsequently, general blocking was performed using bovine serum albumin (BSA) solution and serum fetal bovine (SFB). The slides were incubated overnight in a moist chamber at 4 °C with anti-TMEM160 (1:200 dilution; rabbit IgG; Invitrogen), followed by 1 h of incubation with horseradish peroxidase (HRP)-conjugated secondary antibody at room temperature. A negative control was established by replacing the primary antibody with an antibody dilution solution. The sections were developed with diaminobenzidine tetrahydrochloride and counterstained with hematoxylin. The IHQ was evaluated by a pathologist based on the extent of positivity. The immunohistochemistry staining intensity was interpreted as follows: 1 + (low) if visible with the 400× objective, 2 + (moderate) if visible with the 100× objective, and 3 + (high) if visible with the 40× objective, and immunostaining images were captured using a ZEIZZ Axio Scan Microscope.

### 4.3. Cell Culture

Lung adenocarcinoma cell lines A549, H1975, non-cancer lung cell line BEAS, cervical cancer cell lines HeLa and SiHa, and non-cancer cell line HaCaT were cultured in Roswell Park Memorial Institute (RPMI)-1640 medium with glutamine (Gibco, Grand Island, NY, USA, #11875093). The medium was supplemented with 10% fetal bovine serum, 50 U/mL penicillin, and 50 mg/mL streptomycin (Gibco, Grand Island, NY, USA, #15240-060). Cells were dissociated with 1× trypsin (Gibco, Grand Island, NY, USA, #15400-054), and/or Versen when they reached 70–80% confluence. The dissociated cells were collected for protein extraction.

### 4.4. Protein Extraction

The protein extracts used for proteomic experiments were obtained using a lysis buffer containing 100 mM Tris (pH 8.4) and 4% SDS. Western blotting was performed using lysis buffer NP40 (Invitrogen, Thermo Fisher Scientific, Waltham, MA, USA, #FNN0021) with a protease and phosphatase inhibitor cocktail (Thermo Fisher Scientific, Waltham, MA, USA, #1861281). The cell packages were sonicated in four cycles of 20 s and 30 s in ice between each cycle. NP40 lysis buffer with 0.5% CHAPS and protease inhibitors was used for protein extraction for co-immunoprecipitation. Protein quantification was performed according to each lysis buffer, and protein extracts obtained with SDS were quantified by densitometry of 12% polyacrylamide gels using the GS-800 scanner, whereas extracts obtained with NP40 and NP40 plus CHAPS were quantified with Bradford’s reagent (Bio-Rad, Hercules, CA, USA).

### 4.5. Western Blot Analysis

The following commercially available antibodies were used in this study: anti-TMEM160 (Invitrogen, Waltham, MA, USA, #PA5-49405), goat anti-rabbit IgG-HRP (sc-2030), anti-actin coupled-HRP (sc-1616), anti-lamin A/C (sc-376248), and anti-α-tubulin (sc-32293) all from Santa Cruz Biotechnology, Dallas, TX, USA. Electrophoresis was conducted on 12% and 15% polyacrylamide gels, and the proteins were transferred onto PVDF membranes in a wet system. Membranes were blocked with 5% low-fat milk in Tris-Tween 20 buffered saline solution (TBS-T) for one hour at room temperature with agitation. Subsequently, the membranes were washed three times with TBS-T. The primary antibody was incubated overnight at 4 °C, followed by three washes with TBS-T. The peroxidase-conjugated secondary antibody was incubated for one hour at room temperature. A chemiluminescent substrate (Thermo Fisher Scientific, Waltham, MA, USA, #34580) was used for signal detection, and images were captured using a LICOR C-Digit scanner. Relative quantification was performed using the Image Studio Digits Version 5.2 software.

### 4.6. CRISPR/Cas9 of the TMEM160 Gene and Cell Transfection

A549 cells were transfected with the TMEM160 CRISPR/Cas9 KO (sc-411396), TMEM160 HDR (sc-412396-HDR) plasmids, and control plasmid (sc-418922) from Santa Cruz Biotechnology, Dallas, TX, USA. Transfection was performed using Lipofectamine reagent (Invitrogen, Waltham, MA, USA, #18324-012). Cells were selected 72 h after transfection with puromycin (Santa Cruz Biotechnology, Dallas, TX, USA, sc-108071A). Selected cells were expanded, and single red fluorescent protein (RFP)-positive cells were selected using a FACSAriaFusion cell sorter (Beckman Coulter). Clones were examined by flow cytometry and Western blot to determine TMEM160 depletion.

### 4.7. Cell Proliferation Assay

Cell proliferation was assessed using 3-(4,5-dimethylthiazol-2-yl)-2,5-diphenyltetrazole) (MTT) assay. A total of 2 × 10^3^ cells per well were cultured in 96-well plates with 100 µL of the standard culture medium. Cells were assayed with MTT at 0 h, 24 h, and 48 h. Crystals formed after incubating the cells with MTT for 4 h were dissolved in ethanol and dimethyl sulfoxide vol:vol. The optical density was measured at 595 nm using a microplate reader.

### 4.8. Wound Healing Assay

Approximately 5 × 104 A549 CTRL and A549 KO cells were cultured for 24 h in 6-well plates until they reached 90% confluency. Cells were synchronized 12 h before wounding, leaving them in a medium without fetal bovine serum. A 200 µL micropipette tip was used to wound the cells in each well, and the culture medium was replaced with fresh supplemented medium before being left to incubate. Wound photographs were taken at 0, 12, and 24 h, and the wound closure rate was calculated by measuring the wound area using STcratch software (developed in MATLAB 2008; ETH Zurich).

### 4.9. Transwell Migration Assay

The cell migration assay was conducted using Millicell polycarbonate cell culture inserts (Millipore, Burlington, MA, USA, pore size 8 µm) in 24-well cell culture plates. A total of 5 × 10^4^ A549 CTRL and A549 KO cells were suspended in 200 µL of serum-free medium per well and then placed on top of the polycarbonate insert, and 700 µL of medium supplemented with 10% fetal bovine serum was added to each well to act as a chemoattractant. The culture plates were incubated for 24 h at 37 °C and 5% CO_2_. The non-migrated cells were removed. Cells that migrated through the filter were fixed with 2% formaldehyde for 5 min at room temperature, stained with crystal violet, and counted under a microscope.

### 4.10. Immunofluorescence (IF)

The cells were fixed with 4% formaldehyde for 5 min at room temperature and then permeabilized with 0.05% Triton X-100 for 15 min at room temperature. After that, they were blocked with 5% fetal bovine serum in 1× PBS (phosphate buffered saline) for 1 h at room temperature. The cells were incubated overnight at 4 °C with anti-TMEM160 (Invitrogen, Waltham, MA, USA, #PA5-49405), followed by three washes with PBS. Subsequently, the cells were incubated overnight at 4 °C with anti-α-tubulin (Santa Cruz Biotechnology, Dallas, TX, USA, sc-32293) and washed thrice with PBS. Afterward, the cells were incubated with goat anti-rabbit IgG (H + L) Alexa Fluor 488 (Invitrogen, Waltham, MA, USA, #A-11008) for 1 h at room temperature, followed by five washes with PBS. Then, the cells were incubated with goat anti-mouse IgG (H + L) Alexa Fluor 594 (Invitrogen, Waltham, MA, USA, #A-11008) for 1 h at room temperature and washed five times with PBS. Finally, the cells were stained with 4′,6-Diamidino-2-phenylindole dihydrochloride (DAPI) (Sigma Chemical Co., St. Louis, MO, USA) for 15 min at room temperature and washed thrice with PBS. The coverslips were mounted using Fluoromount-G™ Mounting Medium (Invitrogen, Waltham, MA, USA, #00-4958-02) and examined using a Confocal Olympus FV1000 Upright microscope. The resulting images were with the objective 60× and zoom factor 2. Observations were performed under basal culture conditions without interventions to arrest the cell cycle, allowing for the identification of dividing cells in their natural state. Dividing cells were identified based on morphological criteria: chromatin condensation and mitotic figures visible with DAPI and tubulin staining. In each field of view, between 2 and 4 dividing cells were observed. At least three independent experimental replicates were conducted for each cell line. Images were processed and analyzed using the ImageJ Fiji software (version 2.14.0/1.54f) TMEM160 and tubulin colocalization analysis was performed using the Manders coefficient (M1 and M2). ROIs encompassing a single cell were defined to minimize background areas. The defined ROIs were used in both channels. At least five random regions were analyzed for each cell line. The Manders coefficient was calculated without intensity thresholds to preserve all relevant signals. The results of this analysis are summarized in Table S1 of the Supplementary Materials.

### 4.11. Isolation of Nuclear and Cytoplasmic Extract

Cytoplasmic and nuclear extracts were prepared according to the instructions of the NE-PER Nuclear and Cytoplasmic Extraction Reagent Kit (Pierce Thermo Fisher Scientific, Waltham, MA, USA; #78835). Briefly, cells were washed with PBS and centrifuged at 500× *g* for 5 min. The cell pellet was resuspended by vortexing in 200 µL of cytoplasmic extraction reagent I. The suspension was incubated on ice for 10 min, followed by the addition of 11 µL of the second cytoplasmic extraction reagent II, vortexed for 5 s, incubated on ice for 1 min, and centrifuged for 5 min at 16,000× *g*. The resulting supernatant (cytoplasmic extract) was transferred to a pre-chilled tube. The insoluble pellet fraction containing crude nuclei was resuspended in 50 µL of nuclear extraction reagent by vortexing for 15 s every 10 min for 1.5 h, incubated on ice, and then centrifuged at 16,000× *g*. The resulting supernatant was used as the nuclear extract. All fractions were stored at −70 °C until further use.

### 4.12. Co-Inmunoprecipitation (Co-IP)

Co-IP was performed to purify TMEM160 and its interacting proteins. In short, A549 cells were cultured until 80–90% confluence, the cells were then washed twice with cold 1× PBS and incubated with NP40 lysis buffer (Invitrogen, Waltham, MA, USA, #FNN0021) containing 0.5% CHAPS and a protease and phosphatase inhibitor cocktail (Thermo Fisher Scientific, Waltham, MA, USA, #1861281) for 5 min at 4 °C. Subsequently, the cells were scraped and transferred to Eppendorf tubes. Cells were incubated with gentle shaking for 1 h at 4 °C. The proteins were obtained by centrifugation at 13,500 rpm for 25 min at 4 °C. For co-immunoprecipitation, protein A/G agarose beads (Thermo Fisher Scientific, Waltham, MA, USA, #20421) were incubated with 4 µg anti-TMEM160 antibody (Invitrogen, Waltham, MA, USA, PA5-49405) for 1 h at 4 °C with gentle shaking. Following this, two washes with conjugation buffer (20 mM sodium phosphate) were performed, and the bead-antibody mixture was resuspended with 250 µL of 5 mM BS3 crosslinker, incubated for 30 min at room temperature, and gently homogenized every 5 min. Quenching buffer (1 M Tris-HCl, pH 7.5) was added, and three washes were performed with washing buffer (0.15 M NaCl, 0.028 M Tris base, 0.001 M EDTA, 0.5% NP-40 reagent, 2.5% glycerol). The beads were then resuspended in 1 mg of total protein and incubated overnight at 4 °C with gentle shaking. To eliminate non-specific binding, a negative control was used without the anti-TMEM160 antibody, using 1 mg of total protein and including all other elements of the co-immunoprecipitation. The next day, the supernatant was recovered, and two washes with wash buffer and one wash with 0.15 M saline solution were performed. Bound proteins were eluted from the beads with acidic glycine buffer pH 2.5 and neutralized with 2M tris base pH 11. To assess the immunoprecipitation of TMEM160, 15 µL of the eluate was electrophoresed and resolved on a 15% polyacrylamide gel, and Western blotting was performed using a previously described TMEM160 antibody. Subsequently, the immunoprecipitated complexes were analyzed to identify proteins associated with TMEM160 using liquid chromatography coupled with mass spectrometry (LC-MS/MS).

### 4.13. LC-MS/MS Analysis

The immunoprecipitated proteins were resuspended in 100 mM Tris pH 8.6, reduced with dithiothreitol (DTT), and alkylated with iodoacetamide. Proteins were precipitated with cold ethanol and then digested with trypsin (1:50 enzyme-to-substrate ratio) at 37 °C for 16 h in digestion buffer (50 mM ammonium bicarbonate, 0.5% sodium deoxycholate). After digestion, sodium deoxycholate was removed using ethyl acetate extraction. The samples were desalted with Sep-Pak tC18, and peptides were dried in a vacuum concentrator.

Peptide samples were analyzed using a high-resolution LC-MS system (Dionex Ultimate 3000 RSLCnano UPLC coupled to a Q-Exactive HF-X mass spectrometer) (Thermo Fischer Scientific, Waltham, MA, USA). Data were acquired following a high-resolution variable window DIA method as previously described [60]. Briefly, 1 µg of peptides was loaded and desalated on an Acclaim PepMap100 C18 trap column (3 µm, 100 100 Å, 75 µm i.d. × 2 cm) with aqueous 0.1% TFA at a flow rate of 5 µL/min for 6 min. The separation of peptides was performed in an EASY-spray RSLC C18 analytical column (2 µm, 100 Å, 75 µm i.d. × 50 cm) at a flow rate of 300 nL/min. The temperatures of the trap and analytical columns were set at 35 °C and 60 °C, respectively.

Peptides were separated using a non-linear gradient over 60 min, starting from 2% to 27% buffer B in 55 min, then from 27% to 35% and from 35% to 55% over 2 min each, and finally reaching 90% buffer B in 1 min. The acquisition method was composed of a full acquisition cycle consisting of 54 variable-width MS2 scans and 3 MS1 scans over the mass range of 385–1460 *m*/*z*. The MS2 window width was determined based on the empirical distribution of signals from DDA runs with similar samples, with an overlap of 1 Da between adjacent windows.

The parameters for MS1 were as follows: resolution of 120,000 (@200 *m*/*z*), AGC target of 3 × 10^6^, and maximum injection time of 50 ms. For MS2, the resolution was set to 30,000, with an AGC target of 1 × 10^6^, an automatic maximum injection time, and an NCE of 28.

### 4.14. Protein Identification and Data Analysis

Peptide and protein identification was performed with DIA-NN v1.8.1. The identification of peptides and proteins was conducted library-free (direct mode) using the Uniprot human (2020 version) as the reference. DIA-NN parameters were tailored for optimal peptide and protein identification. The precursor and fragment mass ranges were set between 300–1800 *m*/*z* and 200–1800 *m*/*z*, respectively, with the precursor charge ranging from +1 to +4. Up to 2 missed cleavages were allowed for the tryptic peptides generated in silico. Only peptides between 7 and 30 amino acids were considered for identification. Cysteine residues were carbamidomethylated as a fixed modification, while methionine oxidation was allowed as a variable modification with a limit of up to two variable modifications per peptide. The maximum false discovery rate (FDR) was set to 0.01 at both the precursor and protein levels. Quantification was performed by using fixed-width centers of elution peaks, while interference removal from fragment elution curves was disabled to avoid bias. The mass accuracy optimization was carried out automatically using the first run in the dataset. Highly heuristic protein grouping was applied in protein identification. The matrix with identified proteins and their profiles was subsequently analyzed in Perseus v2.0.10.0 to assess differential protein expression across conditions. Values were log2 transformed and normalized by the median intensity of all protein entries in the sample. The TMEM160 interactome was analyzed using a Student’s *t*-test to compare protein concentrations between Co-Ip and negative control samples. *p*-values were adjusted for False Discovery Rate (FDR) using the Benjamini-Hochberg method, with <0.05 considered significant. Gene Ontology (GO) terms were analyzed with biological process enrichment analysis using Metascape (https://metascape.org/gp/#/main/step1, accessed on 2 June 2024), ShyniGO (http://bioinformatics.sdstate.edu/go/, accessed on 2 June 2024), Kyoto Encyclopedia of Genes and Genomes (https://www.genome.jp/kegg/, accessed on 2 June 2024), Gene Ontology (http://geneontology.org/, accessed on 2 June 2024), to identify cellular processes and signaling pathways. Gene Set Enrichment Analysis (GSEA) (https://www.gsea-msigdb.org/gsea/index.jsp, accessed on 2 June 2024) was performed on Log2-transformed proteomics data from Co-IP and control samples using the GSEA software (UC San Diego Broad Institute). Enrichment analysis was based on curated gene sets from MSigDB, KEGG, Reactome, Biocarta, and WikiPathways, with a significance threshold of *p* < 0.05, and according to GSEA, gene sets with an FDR < 25% are most likely to generate meaningful hypotheses and guide further research while still providing results for all analyzed [61,62] genes. A total of 1000 permutations were performed, using the signal-to-noise ratio for ranking genes, with gene sets restricted to sizes between 15 and 500 genes.

### 4.15. Tumor Xenograft Mouse Model

The experimental procedures involving animals were approved by the Internal Committees for the Care and Use of Laboratory Animals (CICUAL) of INCan. Four experimental groups were established based on the cell line used: A549 CTRL, A549 KD TMEM160, HeLa CTRL, and HeLa KO TMEM160. Each experimental group consisted of 5 mice, for a total of 20 mice for the study. Male NOD-SCID mice aged 10–12 weeks were subcutaneously injected with 2 × 10^6^ A549 KO and A549 CTRL cells, whereas female NOD-SCID mice of the same age were injected subcutaneously with 2 × 10^6^ HeLa KO and HeLa CTRL cells in 0.2 mL of RPMI culture medium. Tumor development and growth were evaluated on a weekly basis. Tumor volume (TV) was evaluated by measuring the longest diameter (a) and shortest diameter (b) using the Attia-Weiss formula: V = (0.4) (a)(b)2. Thirty-three days later, the mice were euthanized in a compressed CO_2_ chamber and autopsied to examine tumor growth. Tumor weight was recorded after resection, and a portion of each tumor was fixed in 4% formaldehyde and embedded in paraffin for subsequent Hematoxylin and eosin (H&E) staining.

### 4.16. Statistical Analysis

Data analysis was based on at least three independent experiments. Quantitative variables were expressed as means and standard deviations, and statistical significance was determined using the Student’s *t*-test, with *p*-value < 0.05 considered significant. Statistical analyses were conducted using GraphPad Prism version 8.0.1.

## 5. Conclusions

In our study, we identified TMEM160 as a critical factor in lung adenocarcinoma (LUAD) and cervical cancer. We demonstrated that endogenous TMEM160 exhibits dynamic localization throughout the cell cycle, appearing in both the nucleus and cytoplasm. This finding suggests that TMEM160 protein probably has a role in mitotic events and that proteins in the TMEM family are not limited to biological membranes; they are also present in various cellular compartments, indicating that their functions may extend beyond membrane-related roles.

Furthermore, we showed that a decrease in the expression of TMEM160 leads to reduced tumor growth in vivo. The interactome analysis of TMEM160 reveals its involvement in multiple cellular processes, including apical junctions, xenobiotic metabolism, glycolysis, epithelial-mesenchymal transition (EMT), regulation of reactive oxygen species, UV response, DNA repair, the p53 pathway, and the mitotic spindle.

These findings highlight the essential role of TMEM160 in the development of these tumor types, underscoring its potential as a biomarker in clinical oncology and opening avenues for further investigations into its functions and mechanisms.

## Data Availability

The mass spectrometry proteomics data are available to the ProteomeXchange Consortium via the PRIDE partner repository with the data set identified. Any additional requests can be directed to the corresponding author.

## Supplementary Materials

The following supporting information can be downloaded at https://www.mdpi.com/article/10.3390/ijms26031097/s1.
