# Supplementary material for: TMEM160 Promotes Tumor Growth in Lung Adenocarcinoma and Cervical Adenocarcinoma Cell Lines
Source: Int J Mol Sci. 2025 Jan 27;26(3):1097. doi: 10.3390/ijms26031097 (PMC11816668; doi:10.3390/ijms26031097)
Supplement: Supplementary file 1 [file ijms-26-01097-s001.zip › ijms-3275404-supplementary.pdf]

Supplementary Materials

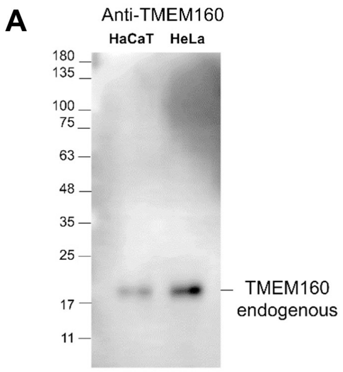

Figure S1. Evaluation of the specificity of the anti-TMEM160 primary antibody.

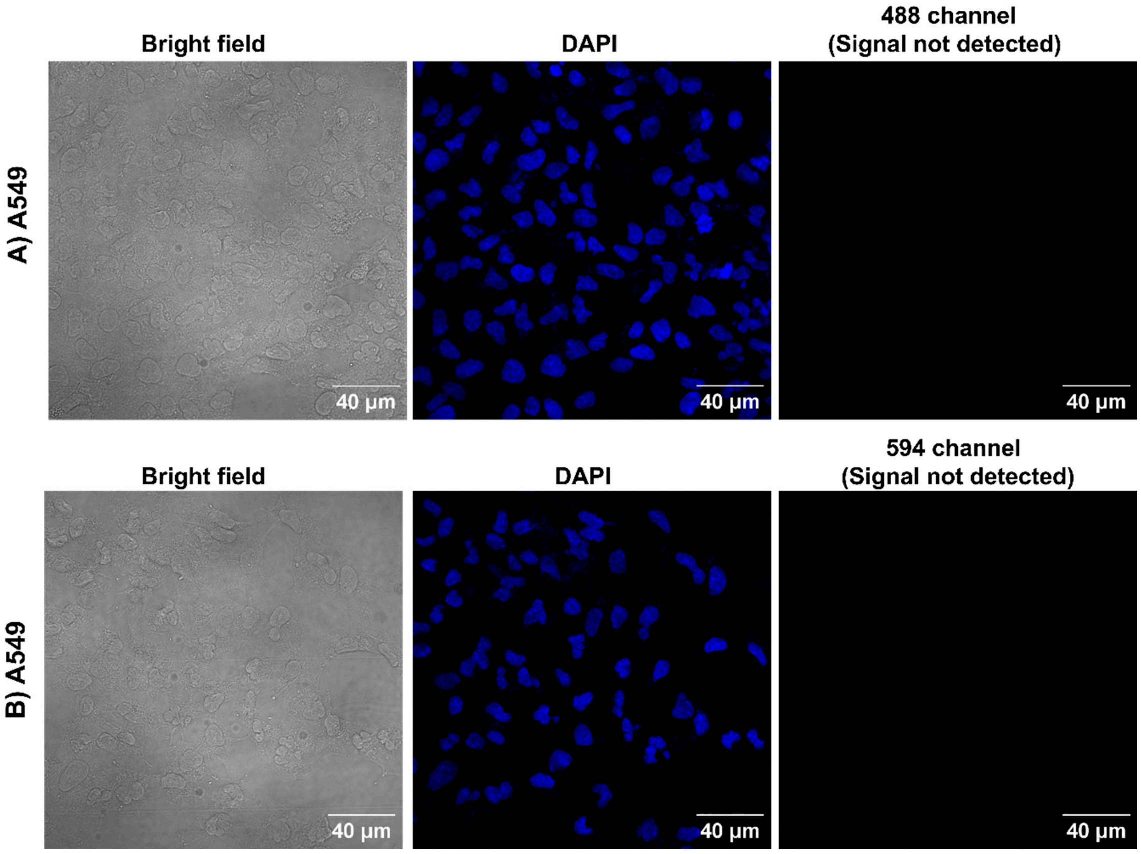

Figure S2. Negative controls without primary antibodies.

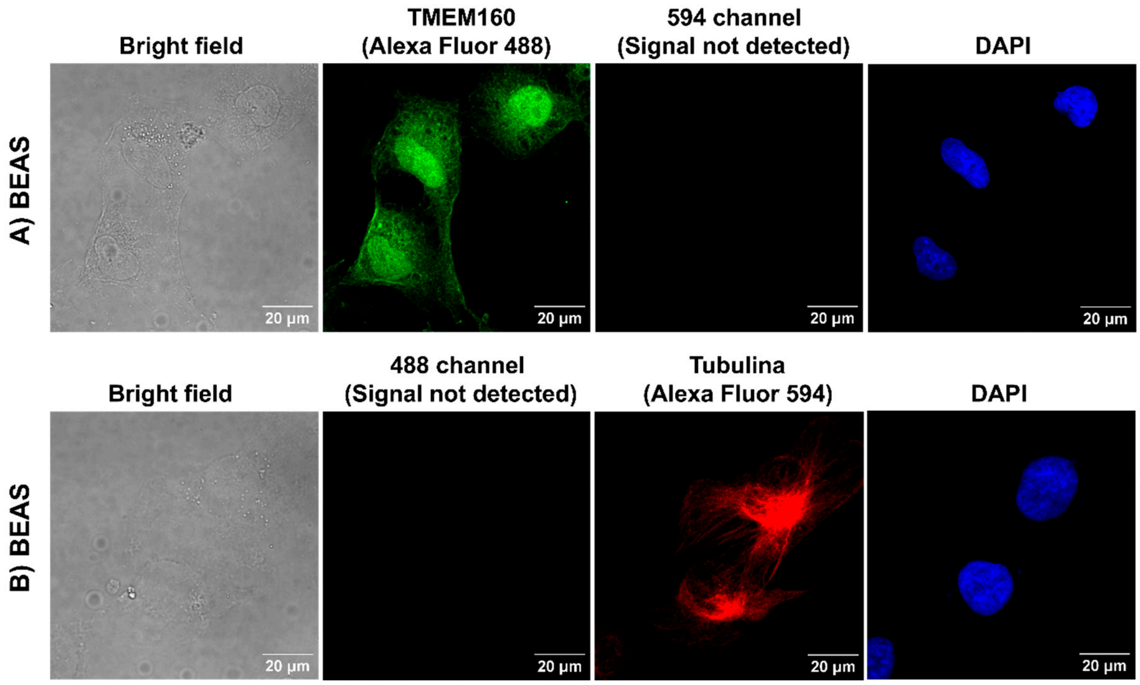

Figure S3. Cross-emission control.

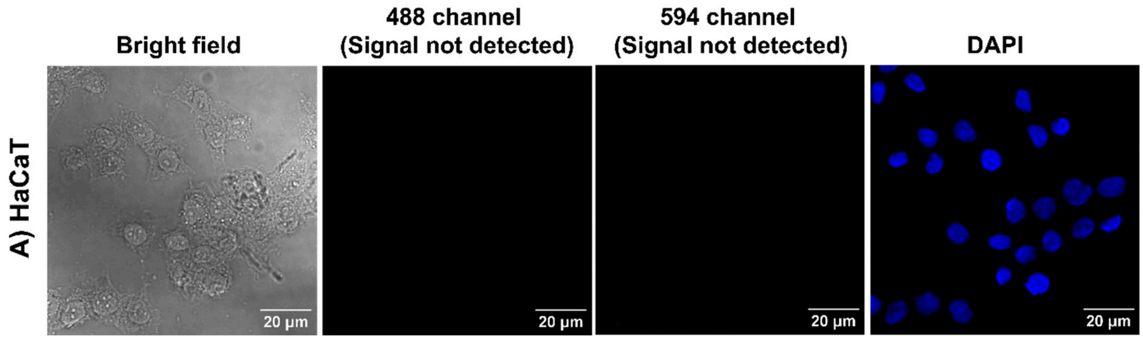

Figure S4. Autofluorescence control.

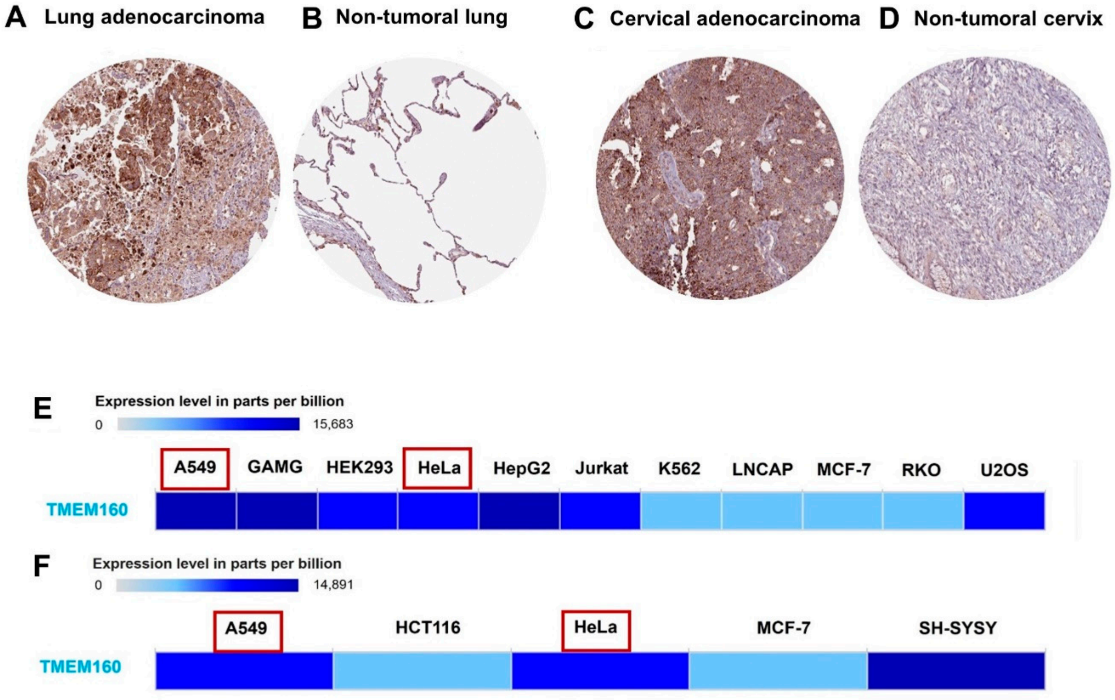

Figure S5. TMEM160 is upregulated in LUAD and cervical cancer cells.

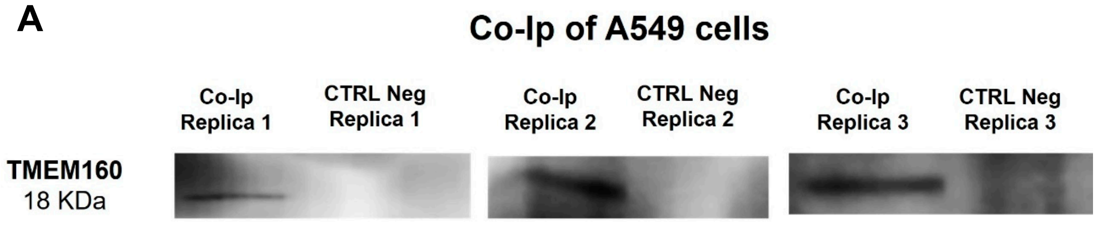

Figure S6. Western blot to assess TMEM160 immunoprecipitation.
